# Improving the Utility of Polygenic Risk Scores as a Biomarker for Alzheimer’s Disease

**DOI:** 10.3390/cells10071627

**Published:** 2021-06-29

**Authors:** Dimitrios Vlachakis, Eleni Papakonstantinou, Ram Sagar, Flora Bacopoulou, Themis Exarchos, Panos Kourouthanassis, Vasileios Karyotis, Panayiotis Vlamos, Constantine Lyketsos, Dimitrios Avramopoulos, Vasiliki Mahairaki

**Affiliations:** 1 Laboratory of Genetics, Department of Biotechnology, School of Applied Biology and Biotechnology, Agricultural University of Athens, 11855 Athens, Greece; dimvl@aua.gr (D.V.); eleni.ppk@gmail.com (E.P.); 2 University Research Institute of Maternal and Child Health and Precision Medicine, and UNESCO Chair on Adolescent Health Care, National and Kapodistrian University of Athens, ‘Aghia Sophia’ Children’s Hospital, 11527 Athens, Greece; fbacopoulou@med.uoa.gr; 3 Center of Clinical, Laboratory of Molecular Endocrinology, Experimental Surgery and Translational Research, Biomedical Research Foundation of the Academy of Athens, 11527 Athens, Greece; 4 Department of Genetic Medicine, Johns Hopkins School of Medicine, Baltimore, MD 21287, USA; rsagar2@jhmi.edu; 5 Bioinformatics and Human Electrophysiology Laboratory, Ionian University, 49100 Corfu, Greece; themis.exarchos@gmail.com (T.E.); pkour@ionio.gr (P.K.); karyotis@ionio.gr (V.K.); vlamos@ionio.gr (P.V.); 6 The Richman Family Precision Medicine Center of Excellence in Alzheimer’s Disease, Johns Hopkins Medicine and Johns Hopkins Bayview Medical Center, Baltimore, MD 21287, USA; kostas@jhmi.edu; 7 Johns Hopkins Medicine and Johns Hopkins Bayview Medical Center, Department of Psychiatry and Behavioral Sciences, Baltimore, MD 21287, USA

**Keywords:** Alzheimer’s disease, polygenic risk scores, biomarkers

## Abstract

The treatment of complex and multifactorial diseases constitutes a big challenge in day-to-day clinical practice. As many parameters influence clinical phenotypes, accurate diagnosis and prompt therapeutic management is often difficult. Significant research and investment focuses on state-of-the-art genomic and metagenomic analyses in the burgeoning field of Precision (or Personalized) Medicine with genome-wide-association-studies (GWAS) helping in this direction by linking patient genotypes at specific polymorphic sites (single-nucleotide polymorphisms, *SNPs*) to the specific phenotype. The generation of polygenic risk scores (PRSs) is a relatively novel statistical method that associates the collective genotypes at many of a person’s SNPs to a trait or disease. As GWAS sample sizes increase, PRSs may become a powerful tool for prevention, early diagnosis and treatment. However, the complexity and multidimensionality of genetic and environmental contributions to phenotypes continue to pose significant challenges for the clinical, broad-scale use of PRSs. To improve the value of PRS measures, we propose a novel pipeline which might better utilize GWAS results and improve the utility of PRS when applied to Alzheimer’s Disease (AD), as a paradigm of multifactorial disease with existing large GWAS datasets that have not yet achieved significant clinical impact. We propose a refined approach for the construction of AD PRS improved by (1), taking into consideration the genetic loci where the SNPs are located, (2) evaluating the post-translational impact of SNPs on coding and non-coding regions by focusing on overlap with open chromatin data and SNPs that are expression quantitative trait loci (QTLs), and (3) scoring and annotating the severity of the associated clinical phenotype into the PRS. Open chromatin and eQTL data need to be carefully selected based on tissue/cell type of origin (e.g., brain, excitatory neurons). Applying such filters to traditional PRS on GWAS studies of complex diseases like AD, can produce a set of SNPs weighted according to our algorithm and a more useful PRS. Our proposed methodology may pave the way for new applications of genomic machine and deep learning pipelines to GWAS datasets in an effort to identify novel clinically useful genetic biomarkers for complex diseases like AD.

## 1. Introduction

Since the human genome was first sequenced, thousands of genetic variants have been associated with biological functions and diseases [1]. Specifically, in regards to diseases, systematic studies called genome-wide association studies (GWAS) have explored the relationship between common sequence variation sites and disease predisposition [2]. The need for GWAS is substantial since most common conditions, such as cardiovascular diseases and cancer, are not caused by a single mutation but are influenced by multiple genetic and environmental factors [3]. GWAS allow the identification of multiple single nucleotide polymorphisms that affect risk for complex diseases [3]. 

Advances in DNA sequencing and the advent of low-priced next-generation sequencing technologies allow many laboratories to apply GWAS to large populations and elucidate the genetic risk factors of multiple diseases. This widespread use of GWAS led to accumulation of immense amounts of data [4] that create various problems in the ability to store and manage information [5]. Bridging the gap from association to actual biological function and filtering GWAS data is not trivial. For example, many variants are co-inherited due to linkage disequilibrium (LD), making it hard to specify which variant is responsible for an association, or, many variants may affect other genes through gene regulation [3].

A common technique to assess disease risk in regards to specific alleles is through the use of polygenic risk scores (PRS) [6]. Polygenic risk scores (PRS) were first introduced in the study of complex disorders as a result of genome wide association studies, a tool that was developed to demonstrate the validity of the results of an otherwise underpowered study on schizophrenia, one of the first GWAS for this disease [7]. While that study, which involved 3322 individuals with schizophrenia and 3587 controls, failed to identify schizophrenia loci with robust statistical support, the authors were able to show the substantial contribution of a “polygenic component” to schizophrenia risk, and that the same component contributed to risk of bipolar disorder [7]. So, PRS offered for the first time the opportunity to explore the consequences of genetic risk variants for one disease on the phenotype, traits and comorbidities of other diseases. Similarly, we have shown that schizophrenia PRS correlates with neurocognitive performance in young adulthood [8]. In later studies, it has also been shown that a polygenic risk score for schizophrenia correlates with comorbid psychosis in Alzheimer’s disease (AD) [9]. It is therefore clear that PRS can be a valuable research tool across many disorders.

The utility of GWAS results, including PRS estimations in clinical practice, has been long debated and questioned. The skepticism has predominantly been a consequence of the small fraction of risk explained both by individual genetic variants and by multiple variants combined in a PRS. However, this appears to be changing. While the total risk variance explained by a PRS is still not very high for any disease, focusing on the extremes of distributions, PRS scores already have significant clinical utility. For example, in a study on coronary artery disease (CAD), Inouye et al. developed a meta-analytic approach to combine large-scale, genome-wide, and targeted genetic association data, and developed a genomic risk score for CAD (metaGRS) with a hazard ratio (HR) for CAD of 1.71 (95% confidence interval [CI]: 1.68 to 1.73) per SD of increase [10]. In this example, those in the top 20% of the metaGRS distribution had a HR of 4.17 (95% CI: 3.97 to 4.38) compared to those in the bottom 20%, an effect comparable to that of *APOE* in AD. MetaGRS was a better predictor for incident CAD than any of six conventional risk factors (smoking, diabetes, hypertension, body mass index, self-reported high cholesterol, and family history) [10]. 

The latest research in PRS calculations has also identified novel risk variants in multifactorial diseases, such as cancer and neurodegenerative diseases. For example, a number of studies have demonstrated breast cancer PRSs as strong risk predictors and have been shown to improve the accuracy of existing risk prediction models, thus implementing them in clinical practice [11,12,13]. The added predictive value of PRS has also been shown by Diana et al. in the case of psychosis risk prediction, where schizophrenia PRS improved risk prediction in non-Europeans [14]. In a different study, Khera et al. developed and validated genome-wide polygenic scores for five common diseases and were able to identify 8.0%, 6.1%, 3.5%, 3.2% and 1.5% of the population that had a greater than threefold increased risk for CAD, atrial fibrillation, type 2 diabetes, inflammatory bowel disease and breast cancer, respectively, which is comparable or better than what one would identify if looking for Mendelian causes of disease [15].

Polygenic risk scores are calculated by first using a reference GWAS to identify variants associated with a disease or phenotype—along with a corresponding statistical confidence, as well as the identity of alleles that increase risk and the odds ratios associated with these risk alleles. Then, for any given individual in an independent dataset one can count the number of risk alleles and weigh them by their effect sizes and log odds ratios to calculate a PRS. This is done after selecting variants exceeding a chosen statistical confidence threshold while commonly many such thresholds are tested to identify the one exhibiting optimal performance. The power of polygenic risk scores depends on the validity of the reference GWAS results which in turn is a function of sample size. 

Fortunately, in recent years through collaborations and meta-analysis, GWAS keep getting larger in sizes and their results are more and more reliable. In the case of AD, a meta-analysis of 17,008 AD cases and 37,154 controls first identified 19 risk loci in addition to the well-known *APOE* [16], and subsequent studies have increased these numbers [17]. Apolipoprotein E (APOE), a major cholesterol carrier that is involved in the regulation of lipid transport, neuronal signaling, and amyloid-beta aggregation [18] and clearance has long been established as a major risk factor for late onset Alzheimer’s disease (LOAD) [19]. The human *APOE* gene is located on chromosome 19 q13.2 and has three common allelic variants, namely ε2, ε3, and ε4 [20]. The corresponding isoforms of the coding protein differ in two positions that differentiate the resulting structure, and thus function, of the lipoprotein [21]. Several studies have shown the isoform-specific *APOE* involvement in the AD pathogenesis and onset of the disease, reporting that *APOE* ε4 allele shows increased levels of amyloid aggregation, lower levels of amyloid clearance due to a non-optimal lipidation state of APOE4 and affects the BBB permeability [22,23,24]. Besides *APOE* ε4 identification, GWAS studies have uncovered more than 20 genetic loci associated with AD risk involved in mainly three pathways, the inflammatory response (CR1, MS4A, CD33, TREM2), lipid metabolism (CLU, ABCA7, SORL1) and endocytosis (BIN1, CD2AP, PICALM) [25,26].

While the sample sizes in the aforementioned studies were larger than what is currently available for AD, they provide proof of principle that as AD GWASs become larger and more reliable, PRS is poised to become of high importance in clinical practice, especially when combined with other risk factors [27]. It has already been shown in postmortem diagnosed sporadic early-onset AD that the predictive ability of identifying cases and controls is better when using PRS than the *APOE* locus alone, with a calculated accuracy of 72.9% and 65.2% respectively when using a standard PRS analysis algorithm, improving to 75.5% in identifying patients when using logistiv regression [28]. It has also been shown that there is a shared genetic architecture between sporadic late onset AD (LOAD) and familial early onset AD, with genetic factors identified for late onset AD also modulating risk in early onset AD cohorts. [29]. 

From the perspective of pathogenesis, PRSs have provided a number of interesting insights into AD. A correlation has been shown between AD PRS and early-life cognition and hippocampal volumes [30], attenuated cerebrovascular function during young adulthood [31], as well as elevated plasma levels of inflammatory biomarkers in AD patients, including complement proteins [32]. Further, a study of mild cognitive impairment (MCI)—a putative AD dementia prodrome—in individuals ≥65 years old reported that a specific PRS can identify individuals at higher risk of conversion to sporadic dementia. 

The above highlights the potential utility of PRSs in Precision Medicine in AD which has been systematically reviewed by Harrison et al. [33]. Logue et al. suggested that PRS can identify MCI in adults who are in their 50s [34]. A study combining patterns of brain atrophy, cognitive scores, *APOE genotype* and CSF biomarkers has highlighted the importance of combining information including genetics for prediction of MCI to AD progression [35], as has already been shown in cardiovascular disease [10]. The combination of a carefully crafted PRS with age, gender and clinical features has great potential in identifying groups of individuals with high risk for AD and pave the way to targeted therapeutics for different groups. Finally, Banks et al. have proposed that PRS can be useful for the design of clinical trials as a stratification factor to identify individuals with underlying AD pathology, making it more efficient, less burdensome on participants, and more cost effective [36].

We believe that the above observations strongly suggest that PRS will become an important tool in studying and characterizing AD. Yet, there is still a significant amount of work remaining for AD PRSs to be strongly included in clinical use for early prediction of AD cases. It is important to keep improving on the power of PRS by increasing the validity of GWAS results. One way to do this is by increasing GWAS sample sizes. Other ways include incorporating other genomic information to select the most reliable genetic associations as we discuss below. Smarter ways to build PRSs, such as constructing pathway-specific PRS have already proven of value. By comparing pathway specific PRS, Tesi et al. proposed that immune response and endocytosis pathways are specifically associated with resilience against AD [37].

As we gain knowledge, PRS will become increasingly powerful (Supplementary Materials Table S1). For example, DeMarco et al. demonstrated that the link between polygenic hazard and neurocognitive variables depends on *APOE*-ε4 allele status [38]. This suggests that clinical phenotypes are influenced by complex genetic interactions. Once we are aware of these complexities, we will be able to increase further the value of PRSs and eventually integrate them as important biomarkers in our efforts to achieve Precision Medicine. As part of this review, we propose an innovative pipeline to produce a novel set of SNPs that improve the PRS calculation and the explained variance through the following analytical steps.

## 2. Approach to Calculating an Improved PRS

Towards the elucidation of the complex genetic associations for multifactorial diseases such as AD, we propose the following pipeline for the likely improvement of PRSs as clinically useful genetic biomarkers with enhanced predictive power. The proposed filtering recalculation for improved PRS is based on the concept of an enhanced annotation and weight application on the single-nucleotide polymorphisms (SNPs) identified in GWAS studies based on their biological function, significance, and phenotypic effect. The proposed methodology aims to overcome three different major drawbacks encountered thus far in the statistical calculation of PRS: co-inheritance and gene-loci dependence, epigenetic effects, and effect on phenotype severity. A major bias in PRS calculation, one that affects discriminative ability, is the coinheritance of multiple risk variants, which results in applying a statistical significance on variants that are not functionally involved in the disease pathology. SNPs identified by GWAS located in protein coding regions have not been differentiated in terms of potential epigenetic effects. Finally, PRS calculations have not accounted for the effect of each variant on the phenotype beyond that calculated by GWAS, in an effort to account for impact on disease severity. 

Below we propose steps to recalculate PRS for AD based on the application of appropriate weights on variants, in an effort to overcome the drawbacks mentioned, above is described:

*Gene loci filtering*: In a GWAS analysis all SNPs that make the threshold on a Manhattan plot are considered important. However, it is possible that some come in batches as they are inherited together. If that is the case then not all of them equally affect the phenotype. So, it is possible that some SNPs score high on PRS without being the causative SNP, but only because they are co-inherited with other causative SNPs. Even though controlling for linkage disequilibrium (LD) is implemented in the PRS calculation, we propose a function-based enhancement to address this bias by taking gene loci filtering into consideration. In conventional PRS calculation, SNPs are thinned by either clumping, which prioritizes among many SNPs in LD in a region based on their p-value, or pruning, which retaining among SNPs in LD those with the largest minor allele frequency (MAF), with the goal to account for LD while capturing the most associated SNPs in a locus [39]. In this approach, we propose an additional refinement following the PRS calculation, a post-LD filtering, where we can further weight each SNP through their chromosomal location in the proximity of AD genes. Genes that have been identified in vitro to contribute to the etiology of a certain disease are assigned a specific ‘bonus’ sore, whilst ensuring that genes that belong to multiple haplotypes do not influence the PRS score calculation. 

*Epigenetic Impact filtering*: In a GWAS Manhattan plot the *x*-axis is the full genome (all autosomal chromosomes). We propose that all SNPs that might be included in an AD PRS be checked on whether are located in or near known genes and in particular in coding regions of annotated genes that code for known proteins or enzymes related to AD, using semantic searches to weight more on genes that are linked to the AD phenotype based on post-translational protein modifications. Specifically, SNPs that belong to coding genes will be investigated on the impact they have on the aminoacid sequence they produce. Using post-translational modification prediction algorithms, it will be possible to score higher those SNPs that could have a significant epigenetic change on a gene product. For example, a SNP that brings in or removes an amino acid residue that gets phosphorylated would have a higher weight. Loss or gain of phosphorylation could account for the phenotype so that taking this into consideration when calculating PRS will improve the weighting approach. SNPs can also have an epigenetic impact depending on their location and it is evident that risk variants are enriched in regions with regulatory elements [40]. Coding and non-coding variants are highly associated with chromatin structure and histone modifications, with coding SNPs enriched at nucleosomes and associated with repressive histone modifications [41,42]. Still, non-coding risk variants identified in GWAS studies can have a diverse functional role such as regulatory elements [43]. In our approach, will also propose for an additional weight based on the effect of non-coding variants on gene expression, by focusing on overlap on open chromatin data and on SNPs that are eQTLs, implementing a variety of computational tools that have been developed to annotate as well as predict such impact [44]. Through this inclusion, specific variants in putative regulatory regions and expression quantitative trait loci associated with the disease will be of higher importance based also on specific tissue expression (brain tissue and nervous system cells).

*Phenotype severity effect:* In many cases and diseases, multiple GWAS studies are available. We propose that a binary classification of severe vs. less severe clinical manifestation for each GWAS be taken into consideration. For example, in AD the unique SNPs that belong to the late onset GWAS study are less potent in causing AD than those in the full AD GWAS dataset that are supposed to be uniquely linked to more severe disease manifestations. Under this logic, genes can be clustered in three groups: those that belong to both datasets (severe and less severe) in regard to their phenotypic influence; those that are unique to the severe phenotype; and those unique to the less severe phenotype. The weights of these SNPs are adjusted accordingly with the main focus on giving extra weight and significance to those SNPs that cause the most severe phenotype. 

**Figure 1:**
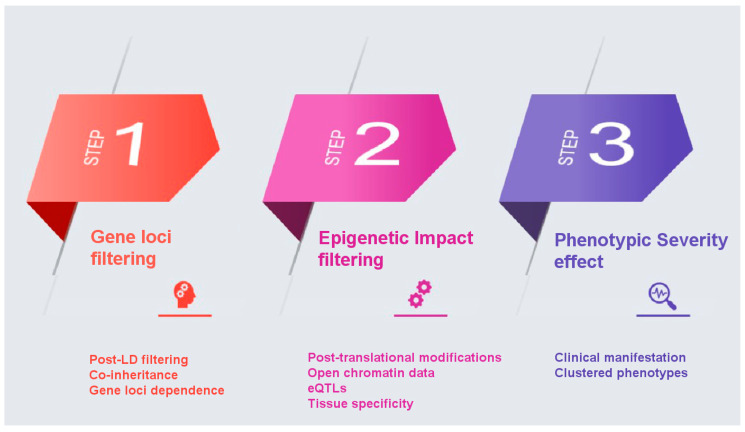
A step-by-step illustration of the proposed filtering pipeline.

*Summary of proposed approach* (Figure 1): We propose to use filtering methods to remove the aforementioned drawbacks that affect the overall calculation of PRS. The first step takes into consideration the inheritance network/relationship between SNPs in areas in the proximity of AD genes, as there might be SNPs that come together with driver/important SNPs because they are linked or inherited together, while not affecting the phenotype. The second step weights more highly SNPs in coding genes because of changes they bring to the polypeptide chain could have post-translational modification impact and an additional weighting system that will control for chromatin accessibility and histone modification. The third step weights more highly SNPs that are associated with more severe forms of the phenotype, when derived from different GWAS studies, as illustrated above with AD. 

### Laying the Foundation for Applying the above Approach to Estimate PRS in AD

While our described approach remains theoretical until actual analyses are performed and benchmarked, to better describe the approach we will describe steps we have taken towards this direction. We have developed a dataset that integrates all Alzheimer’s Disease (AD) related genes, proteins and SNP associations reported in the literature. A general-purpose pipeline has been designed that combines list of genes associated with AD, available either in gene or protein databases. First, the complete list of 854 AD related genes was downloaded from the NCBI gene database using the keywords “Alzheimer’s Disease” [45]. We filtered this list of genes with respect to species and retained only entries regarding Homo Sapiens. Due to significant redundancy in terms of included attributes, we applied filtering to isolate only the attributes of interest: Gene ID, Symbol, Aliases, Map location, Chromosome. Second, to enhance the gene dataset, a list of 6965 AD related proteins was downloaded from the NCBI protein database under the same keyword “Alzheimer’s Disease” [46]. The accession numbers were retained and an SQL script was developed to automatically scan the UniProt database and extrapolate the corresponding gene name (Gene ID) using the online converter of UniProt [47]. The consequent attributes for each entry were appended to the protein dataset and entries were also filtered by species and duplicates were removed. Third, both lists were imported into MySQL databases with the corresponding attributes, originating from the AD related gene dataset and the AD related protein dataset. Comparing the two datasets, if a gene corresponding to a specific protein was already included in the gene dataset, it was annotated with the corresponding protein by including one additional attribute. If the specific gene (coming from the list of proteins) was not included, a relevant entry was appended in the file, thus enhancing the original list of genes related to Alzheimer’s Disease. 

An enhanced non-redundant dataset was thus developed, including all AD related genes originating either from the protein dataset or from the gene dataset. The dataset consists of 630 entries with the following attributes: *Gene ID*, *Symbol*, *Map_location*, *Chromosome*, *Origin.* The field *Origin* was annotated with three distinct values: (a) *common*, which annotates all genes that existed both at the gene dataset and the protein dataset (in total 54 common genes), (b) *GenesAD*, which annotates all genes that were included only in the original gene dataset (in total 496 genes), and (c) *ProteinsAD*, which annotates all genes that were included only in the protein dataset (in total 80 genes). The full list of AD genes is in Supplementary Materials Table S2.

To complete the annotation of the dataset, the set of AD related GWAS studies was downloaded from the GWAS Catalog [48]. The SNPs identified with an association to AD were compared to the in-house gene dataset and an additional attribute was appended in terms of their corresponding genes. 144 unique entries with the corresponding genes of the GWAS catalog were identified and included in the in-house dataset. Out of these, 42 genes originating from the AD related protein dataset was also appended with the amino acid change involved. The latter datasets can be found in Supplementary Materials Tables S3 and S4, respectively.

The GWAS dataset used contains two distinct traits for Alzheimer’s Disease: late-onset Alzheimer’s disease (EFO_1001870) and Alzheimer’s disease (EFO_0000249). The main concept is that late-onset Alzheimer’s disease SNPs are associated with a less severe clinical form of AD. SNPs from both studies were mapped on the 1241 AD related genes that we identified using the data collection pipeline. This resulted in three groups of SNPs based on their overlap in the two GWAS datasets. One group includes SNPs that are identified in both the Alzheimer’s disease (EFO_0000249) dataset and the late-onset Alzheimer’s disease (EFO_1001870) dataset and are given neither bonus nor penalty. One group that includes SNPs that are unique to the Alzheimer’s Disease (EFO_0000249) GWAS study only and consecutively were given a bonus score (as they belong to the severe clinical manifestation group). And one group that includes those SNPs that are unique in the late-onset AD (EFO_1001870), which were accordingly given a score penalty as they are linked with a milder manifestation of AD. 

We are optimistic that our planned future analyses will confirm that our approach can significantly enhance the performance of PRSs and subsequently their utility in the clinic.

## 3. Conclusions

While very significant progress has been made, and work on other diseases suggests the PRS analysis is likely to become an important tool for precision medicine, the current state of PRS in AD is not yet where it would need to be for this purpose. Although numerous studies have been conducted, AD PRSs’ predictive power are not satisfactory for clinical use. The overall calculation of PRSs is highly dependent on the sample sizes of GWAS studies and the genetic architecture of the disease, where more dense genotyping and larger number of SNPs enhance their predictive power but on the other hand increase noise in effect size estimates. The variety of computational methods used for PRS construction and validation, inclusion of additional variables to adjust for risk prediction, linkage disequilibrium, as well as diversity of the population samples genotyped in different GWAS studies, although useful for achieving improved genetic risk associations, comprise a drawback in the replicability of predictions and a standardized PRS construction to be applied for clinical use. Given the need for early prediction and the diversity of AD clinical manifestation and genetic background, we propose the improvement of a PRS calculation refined by additional weighting based on an extensive annotation of SNPs and their biological function that will reformulate their effect on the disease.

Using a data mining and data fusion pipeline we propose to establish a consensus PRS of AD risk based on the inheritance pattern of SNPs, the post-translational impact of SNPs (where applicable) and the associated phenotypic trait (AD = severe; late AD = less severe). We argue that this novel PRS will more efficiently represent the pathogenic impact of multiple gene variants on AD phenotypes—and on other multifactorial diseases. The first step of our proposed filtering is most crucial for noise reduction as it excludes co-inherited variants that do not affect the phenotype under study while reducing dataset size. The steps of post-translational modification filtering and phenotype severity impact introduce additional features that should be included in the PRS calculation but require for appropriate weight assignment. To assess the effects of variants in coding regions on post-translational modification we determined whether AD GWAS variants correspond to genes included in the AD-related protein dataset. We annotated variants as “AD-related” when the change they introduced was missense resulting in an aminoacid change. Aminoacid changes are more likely to be functional as they can affect the structure and/or function of the protein, whether in terms of physical or biochemical properties. Also, if an aminoacid change occurs at a post-translational modification site it could have a profound effect on signaling. Thus, accounting for such information will aid assignment of an appropriate PRS weight.

The effect of gene variants on phenotype severity feature can be approached in multiple ways, depending on the disease studied. In the case of AD, we divided the dataset into two groups: the late-onset versus early-onset AD, as annotated in the GWAS catalog, and applied a simple bonus and penalty scoring system to clearly discriminate this phenotypic effect. In other diseases, especially where data are available to classify phenotypic severity into more than two groups, the scoring system might be more complex. Once these features are determined the proposed risk score can be calculated. This approach will overall filter out miscalculated SNPs that were highly associated with severe disease risk due to noise effect sizes, co-inheritance and gene-loci dependence and allow for a new set of SNPs to surpass the given threshold based on their actual effect on gene expression, post translational modifications, and phenotypic severity.

Given the complexity of AD and the multidimensionality of the underlying genetics future efforts will be focused on establishment of an advanced mathematical function to correlate the genetic profile of each patient to the phenotype and severity of the disease. The methodology we propose is expected to filter out false-positive SNPs in conventional GWAS analyses and lead to a more precise and reliable SNP subset with actual potential to be used as an AD biomarker. Incorporating clinical data in the PRS construction methodology can enhance the predictive ability of a PRS score with a clinical benefit. So far, the construction of a purely statistical, GWAS-based PRS has shown limited applicability. The optimization of a risk model should include empirical data and additional machine learning methods in a personalized manner, that will include variables not only based on the molecular mechanisms and genetic architecture of the underlying disease, but also interpret the effect of population origin, comorbidities, clinical data on the disease phenotype, and a medical profiling of the groups under study, under the scope of the probable epigenetic effects for each patient. Acknowledging the current limitations of a personalized PRS score, the proposed approach can refine the risk model based on the clinical manifestation of AD. The validation of our model can be achieved by additional GWAS studies in large cohorts that will include a more precise description on the phenotypic severity, as well as future clinical outcomes for each person, expanding the input data for our simulations with additional features and empowering the predictive outcome of machine learning simulations.

In conclusion, we propose that this hybrid filtering and scoring pipeline can be applied in more GWAS studies and diseases and act as a mathematical algorithm capable of grouping patients in phenotypic groups, therefore serving as an in-silico clinically useful biomarker of disease risk and lay the groundwork for an optimized PRS construction method. Refining the proposed pipeline will require artificial intelligence methods, such as deep learning, applied to GWAS datasets, and enhanced phenotyping, perhaps through medical records, to calibrate weights of each variant on the PRS calculation. The combined approaches of machine learning, enhanced with clinical data, and PRS can further improve the predictive capability of an improved risk score for multifactorial diseases.

## Data Availability

The data presented in this study are openly available in NCBI gene database (https://www.ncbi.nlm.nih.gov/gene/) (accessed on 28 June 2021), NCBI protein database (https://www.ncbi.nlm.nih.gov/protein/) (accessed on 28 June 2021), and NHGRI-EBI GWAS catalog (https://www.ebi.ac.uk/gwas/) (accessed on 28 June 2021).

## Supplementary Materials

The following are available online at https://www.mdpi.com/article/10.3390/cells10071627/s1, Table S1: List of Studies used in the calculation of PRS in Alzheimer’s disease; Table S2: AD related genes annotated by origin dataset; Table S3: List of 144 unique entries of genes identified in AD GWAS catalog and the in-house database; Table S4: List of the 42 genes originating from the AD related protein dataset.
