# Supplementary material for: Improving the Utility of Polygenic Risk Scores as a Biomarker for Alzheimer’s Disease
Source: Cells. 2021 Jun 29;10(7):1627. doi: 10.3390/cells10071627 (PMC8305482; doi:10.3390/cells10071627)
Supplement: Supplementary file 1 [file cells-10-01627-s001.zip › cells-1183159 Sm/SuppTable 1.pdf]

Sup. Table 1. List of Studies used in the calculation of PRS in Alzheimer's disease

Author /Year	Method of calculating the score/weighting the components	Component used to construct PRS/PHS	SNPs or alleles included when calculating the PRS/PHS	Index of association (RR, OR, HR) between the PRS and the outcome
Sleegers K, *et al*. 2015	Summation of risk alleles per individual was used to calculate counted genetic Risk score (cGRS).<br><br>Strength of the genetic association (wGRS) was derived each allele of every SNP with their OR an sum of these SNPs per sample.<br><br>The GWAS data of independent European ancestry was used to weight the simple effect of APOE e4 to GRS.<br><br>APOE was based on age-specific ORs for different APOE e2/3/4 to see the association of onset of age.<br><br>Participant excluded if data found to be missing	22 SNP (APOE) found to be major, age onset, family history and level of CSF-Aβ42<br><br>IGAP meta-analysis loci excluding DSG2 plus APOE, TREM2	APOE rs7412/ rs429358<br>CR1 rs3818361<br>BIN1 rs744373<br>CD2AP rs9349407<br>ABCA7 rs3764650<br>HLA-DRB5 rs9271192<br>PTK2B rs28834970<br>INPP5D rs35349669<br>CELF1 rs10838725<br>FERMT2 rs17125944<br>TREM2 rs75932628<br>EPHA1 rs11767557<br>CLU rs11136000<br>MS4A6A rs610932<br>PICALM rs3851179<br>CD33 rs3865444<br>SORL1 rs11218343<br>SLC24A4 rs10498633<br>MEF2C rs190982<br>NME8 rs2718058<br>ZCWPW1 rs1476679<br>CASS4 rs7274581	OR
Mormino EC, *et al*. 2016	Summation of reference allele counts at each SNP weighted by the log OR at stage 1.<br><br>Independent loci were identified using PLINK's linkage disequilibrium (LD) clumping procedure, which reveals correlated sets of SNPs.<br><br>19 SNP that were significant in the IGAP meta-analysis were included and analyses controlled for APOE4, age, and sex, as well as 5 principal components from a multidimensional scaling analysis to account for population heterogeneity<br><br>Stage I IGAP SNPs $p < 0.01$ (this threshold was chosen due to its ability to discriminate between AD and controls on ADNI sample.)<br><br>Cognitive decline, clinical progression, hippocampus volume and Aβ in older participant with dementia	19 SNP that were significant in the IGAP meta-analysis were included and analyses controlled for APOE4, age, and sex, as well as 5 principal components<br><br>PGRS and hippocampus volume examined in healthy younger participants (age 18–35 years).	rs4844610 CR1<br>rs6733839 BIN1<br>rs10933431 INPP5D<br>rs190982 MEF2C<br>rs9271192 HLA-DRB5/HLA-DRB1<br>rs10948363 CD2AP<br>rs2718058 NME8<br>rs1476679 ZCWPW1<br>rs11771145 EPHA1<br>rs28834970 PTK2B<br>rs9331896 CLU<br>rs10838725 CELF1<br>rs983392 MS4A6A<br>rs10792832 PICALM<br>rs11218343 SORL1<br>rs17125944 FERMT2<br>rs10498633 SLC24A4/RIN3<br>rs4147929 ABCA7<br>rs7274581 CASS4	OR

	Older CN participants from ADNI had Mini-Mental State Examination (MMSE) score24, Clinical Dementia Rating (CDR) 0, and were within the normal range on education-adjusted Logical Memory Delayed recall cutoffs.			
Chouraki V, *et al.* 2016	Derived weighted sum of risk alleles from the 19 top SNPs reported by the IGAP GWAS in participants aged 65 and older without prevalent dementia<br>18 SNP-based GRS was computed excluding APOE.<br><br>Calculated: C-index, NRI and IDI to see whether the incremental improvement<br><br>IGAP meta-analysis loci, excluding APOE, DSG2, and CD33<br><br>A base model containing age, sex, education, and APOE ε4.<br><br>PRS models for age at baseline, sex, education levels, and presence or absence of at least one APOE ε4 including GRS.	18 SNPs, age, Sex and Education	rs4844610 CR1<br>rs6733839 BIN1<br>rs10933431 INPP5D<br>rs190982 MEF2C<br>rs9271192 HLA-DRB5/DRB1<br>rs10948363 CD2AP<br>rs2718058 NME8<br>rs1476679 ZCWPW1<br>rs11771145 EPHA1<br>rs28834970 PTK2B<br>rs9331896 CLU<br>rs10838725 CELF1<br>rs983392 MS4A6A<br>rs10792832 PICALM<br>rs11218343 SORL1<br>rs17125944 FERMT2<br>rs10498633 SLC24A4/RIN3<br>rs4147929 ABCA7<br>rs7274581 CASS4	HR
Escott-Price V, *et al.* 2015	SNPs w/AD association p < 0.5 within IGAP samples excluding<br><br>GERAD data was used to compute the PRS<br><br>AUC after inclusion of ε4, ε2, age, sex, 20 IGAP GWAS SNPs, and SNPs w/AD association p< 0.5.<br><br>Predictive modelling was performed using a polygenic score approach based upon AD associated SNPs according to the IGAP study (19-SNPs) after excluding the APOE	A total 24 SNPs used to<br><br>PRS with SNPs p≤0.5 APOE region excluded to adjust confounding affect	CR1 rs6656401<br>BIN1 rs6733839<br>INPP5D rs35349669<br>MEF2C rs190982<br>CD2AP rs10948363<br>NME8 rs2718058<br>ZCWPW1 rs1476679<br>EPHA1 rs11771145<br>PTK2B rs28834970<br>CLU rs9331896<br>TP53INP1 gene-based GWAS<br>CELF1 rs10838725<br>MS4A6A rs983392<br>PICALM rs10792832<br>SORL1 rs11218343<br>FERMT2 rs17125944<br>SLC24A4/RIN3 rs10498633<br>IGHV167 gene-based GWAS<br>DSG2 rs8093731<br>ABCA7 rs4147929<br>APOE e4/e2<br>CD33 rs3865444<br>CASS4 rs7274581<br>HLA-DRB5/DRB1rs9271192	OR
Desikan RS, *et al.* 2017	Stepwise regression used to select SNP within ADGC Phase 1 &2 cohort<br>Analysed IGAP data set by using Cox stepwise regression<br>Calculated PHS using 31 stratified SNPS.	31-SNPs and Age	APOE rs429358/rs7412<br>CR1 rs4266886/rs61822977<br>BIN1 rs6733839<br>INPP5D rs10202748<br>HLA-DRB5 rs115124923<br>HLA-DQB1 rs115675626	log HR

			GPR115 rs1109581<br>BC043356 rs17265593/rs2597283<br>ZCWPW1 rs1476679<br>AL833583 rs78571833<br>PTK2B rs12679874<br>CHRNA2 rs2741342<br>CLU rs7831810/rs1532277/rs9331888<br>CR595071 rs7920721<br>SPI1 rs3740688<br>MS4A6A rs7116190<br>PICALM rs526904/rs543293<br>SORL1 rs11218343<br>FERMT2 rs6572869<br>SLC24A4 rs12590273<br>ab Parts rs7145100<br>TRIP4 rs74615166<br>BZRAP1 rs2526378<br>C19orf6 rs117481827<br>ABCA7 rs7408475/rs3752246<br>CASS4 rs7274581	
Rojas ID, *et al.* 2019	PRS were generated by multiplying the genotype dosage of each risk allele for each variant by its respective weight, and then summing across all variants.<br><br>Independent data was validated (assessing effects of subthreshold signal, diagnostic certainty, age at onset and sex) and tested its effect on risk and age at onset in the GR@ACE/DEGESCO study.	39 SNPs, age and Sex	rs4844610 CR1<br>rs876461 PRKD3<br>rs6733839 BIN1<br>rs10933431 INPP5D<br>rs4351014 HS3ST1<br>rs9275152 HLA-DRB1<br>rs143332484 TREM2<br>rs75932628 TREM2<br>rs9381040 TREML2<br>rs9381564 CD2AP<br>rs1859788 PILRA<br>rs56402156 EPHA1<br>rs73223431 PTK2B<br>rs9331896 CLU<br>rs34674752 SHARPIN<br>rs34173062 SHARPIN<br>rs7920721 ECHDC3<br>rs3740688 SPI1<br>rs1582763 MS4A2<br>rs3851179 PICALM<br>rs11218343 SORL1<br>rs17125924 FERMT2<br>rs11623019 RIN3/SLC2A4<br>rs593742 ADAM10<br>rs117618017 APH1B<br>rs7185636 IQCK<br>rs4985556 IL34<br>rs12444183 PLCG2<br>rs3935877 PLCG2<br>rs72824905 PLCG2<br>rs72835061 CHRNE<br>rs75511804 SCIMP<br>rs2732703 KANSL1<br>rs2732703 KANSL1<br>rs616338 ABI3<br>rs4311 ACE<br>rs3752231 ABCA7<br>rs12459419 CD33<br>rs6024870 CASS4	OR

			rs2154481 APP	
Tosto G, *et al.* 2017	The association between the GRS and LOAD was studied in separate age- and sex-adjusted generalized mixed logistic regression models that included the ascertainment site (ADC center) as well as the family as random effects to adjust for possible center variability and intrafamilial correlations	LOAD ~ SEX + AGE + GRS<br><br>LOAD ~ SEX + AGE + APOE-e4 + GRS<br><br>LOAD ~ SEX + AGE + APOE-e4 + GRS + GRS*APOE-e4	rs6656401 CR1<br>rs6733839 BIN1<br>rs35349669 INPP5D<br>rs190982 MEF2C<br>rs9271192 HLA-DRB5/1<br>rs10948363 CD2AP<br>rs2718058 NME8<br>rs1476679 ZCWPW1<br>rs11771145 EPHA1<br>rs28834970 PTK2B<br>rs9331896 CLU<br>rs10838725 CELF1<br>rs983392 MS4A6A<br>rs10792832 PICALM<br>rs11218343 SORL1<br>rs17125944 FERMT2<br>rs10498633 RIN-SLC24A4<br>rs8093731* DSG2<br>rs4147929 ABCA7<br>rs3865444* CD33<br>rs7274581 CASS4	OR
Cruchaga C, *et al.* 2018	Data was used from the participants of KnightADRC study, DIAN study, ADNI study and NIA-LOAD study.<br><br>Weighted PRS, modeling the odd ratios (ORs) as reported in IGAP study using a logarithm of base 2 transformation. SNPs utilized for the score would either need to have a high genotyping rate (greater than 90%) or otherwise be a reasonable proxy to the IGAP hits.<br><br>Polygenic risk scores (PRSs) were constructed using 21 genome-wide significant loci identified for sLOAD	21 SNPs	BIN1 rs4663105<br>CLU rs9331896<br>ABCA7 rs3764650<br>CR1 rs6656401<br>PICALM rs10792832<br>MS4A6A rs983392<br>CD33 rs3865444<br>CD2AP rs10948363<br>EPHA1 rs11771145<br>HLA-DRB5—1 rs111418223<br>PTK2B rs28834970<br>SORL1 rs11218343<br>SLC24A4 RIN3 rs10498633<br>DSG2 rs8093731<br>INPP5D rs35349669<br>MEF2C rs190982<br>NME8 rs2718058<br>ZCWPW1 rs1476679<br>CELF1 rs10838725<br>FERMT1 rs17125944<br>CASS4 rs113902203	OR
Yokoyama JS, *et al.* 2015	Individuals 65- to 101-years-old (N = 216 males, N = 232 females) were evaluated at the University of California, San Francisco Memory and Aging Center (UCSF MAC) and had genotype data available for analysis.	21 SNPs, age, Sex and Education	APOE rs429358/rs7412<br>HFE rs1799945<br>PICALM rs3851179<br>CR1 rs6701713<br>SLC6A4 rs2020942<br>TPH1 rs1799913<br>KIAA0319 rs4504469<br>CDC42BPA rs1320490<br>TMEM175 rs6599389<br>SORL1 rs2070045<br>CNTNAP2 rs17236239<br>ATP2C2 rs8053211<br>CD2AP rs9349407<br>TPD52 rs7814569<br>COMT rs4680	OR

			C9ORF72 rs3849942<br>CPE rs11186856<br>SORL1 rs12285364<br>RIT2 rs4130047<br>MOBP rs1768208	
Marden JR, *et al*. 2016	IGAP meta-analysis loci plus APOE<br><br>Calculated the AD-GRS by multiplying each individual's risk allele count for each locus by the beta coefficient for that polymorphism.<br>Summing the products for all 22 loci.<br><br>This step essentially weighted each polymorphism in proportion to its anticipated effect on dementia risk. Next, to convert to the odds of dementia for each individual, exponentiated the weighted allele sum, multiplied the resulting value by 0.1 and converted odds to probabilities.	22 SNPs, age, Sex, race and Education<br><br>Validated GRS with/without APOE in Dementia	APOE rs429358/rs7412<br>BIN1 rs4663105<br>CLU rs9331896<br>ABCA7 rs3764650<br>CR1 rs6656401<br>PICALM rs10792832<br>MS4A6A rs983392<br>CD33 rs3865444<br>CD2AP rs10948363<br>EPHA1 rs11771145<br>HLA-DRB5/1 rs111418223<br>PTK2B rs28834970<br>SORL1 rs11218343<br>SLC24A4 RIN3 rs10498633<br>DSG2 rs8093731<br>INPP5D rs35349669<br>MEF2C rs190982<br>NME8 rs2718058<br>ZCWPW1 rs1476679<br>CELF1 rs10838725<br>FERMT1 rs17125944<br>CASS4 rs113902203	log OR
Leonenko G, *et al*. 2018	To evaluate the contribution of the 25 SNPs over and above APOE e2 and e4 risk alleles, PHS and PRS were derived in three ways (1) only using e2 and e4 risk alleles, (2) 25 SNPs, and (3) combining e2, e4, and 25 SNPs.<br><br>Tested whether the addition of either PHS or PRS into the Cox regression model improves the model fit over and above APOE e4 and e2 risk alleles using Anova function in R.<br><br>Since APOE is the strongest predictor of AD risk, they also validated the results in e3 homozygous individuals (N = 4368).<br><br>Furthermore, to investigate the stability of the results o for the 25 SNPs of interest, this analysis was repeated for randomly selected subsets of cases and controls.<br><br>IGAP and GERAD data was used for the analysis	31 SNP for the PRS and PHS	APOE rs429358/rs7412<br>CR1 rs4266886/rs61822977<br>BIN1 rs6733839<br>INPP5D rs10202748<br>HLA-DRB5 rs115124923<br>HLA-DQB1 rs115675626<br>GPR115 rs1109581<br>BC043356 rs17265593/rs2597283<br>ZCWPW1 rs1476679<br>AL833583 rs78571833<br>PTK2B rs12679874<br>CHRNA2 rs2741342<br>CLU rs7831810/rs1532277/rs9331888<br>CR595071 rs7920721<br>SPI1 rs3740688<br>MS4A6A rs7116190<br>PICALM rs526904/rs543293<br>SORL1 rs11218343<br>FERMT2 rs6572869<br>SLC24A4 rs12590273<br>ab Parts rs7145100<br>TRIP4 rs74615166<br>BZRAP1 rs2526378<br>C19orf6 rs117481827<br>ABCA7 rs7408475/rs3752246<br>CASS4 rs7274581	OR and HR
Lupton MK, *et al*. 2017	Five data set were used to calculate PRS (ADNI, AddNeuroMed, OATS, SydneyMAS and QTIM)	19 SNPs, age, sex, and 4 ancestry principal components	SORL1 rs11218343<br>BIN1 rs6733839<br>CR1 rs6656401<br>CLU rs9331896<br>PICALM rs10792832<br>ABCA7 rs4147929	OR

	A PRS was constructed from genome-wide SNP array data using 19 genome-wide significant AD risk variants.<br><br>Scores were calculated by summing the number of risk alleles weighted by the effect size.		CASS4 rs7274581<br>FERMT2 rs17125944<br>EPHA1 rs11771145<br>MS4A6A rs983392<br>HLA-DRB5/DRB1rs9271192<br>SLC24A4/RIN3 rs10498633<br>ZCWPW1 rs1476679<br>CD2AP rs10948363<br>PTK2B rs28834970<br>MEF2C rs190982<br>NME8 rs2718058<br>CELF1 rs10838725<br>INPP5D rs35349669	
Tan CH, *et al.* 2018	Each ADNI and ROSMAP participant in this study been calculated for their individual PHS	39 SNPs, age to calculate PHS	APOE rs429358/rs7412<br>CR1 rs4266886/rs61822977<br>BIN1 rs6733839<br>INPP5D rs10202748<br>HLA-DRB5 rs115124923<br>HLA-DQB1 rs115675626<br>GPR115 rs1109581<br>BC043356 rs17265593/rs2597283<br>ZCWPW1 rs1476679<br>AL833583 rs78571833<br>PTK2B rs12679874<br>CHRNA2 rs2741342<br>CLU rs7831810/rs1532277/rs9331888<br>CR595071 rs7920721<br>SPI1 rs3740688<br>MS4A6A rs7116190<br>PICALM rs526904/rs543293<br>SORL1 rs11218343<br>FERMT2 rs6572869<br>SLC24A4 rs12590273<br>ab Parts rs7145100<br>TRIP4 rs74615166<br>BZRAP1 rs2526378<br>C19orf6 rs117481827<br>ABCA7 rs7408475/rs3752246<br>CASS4 rs7274581	HR
Adams HH, *et al.* 2015	Genetic risk scores were constructed by multiplying the number of risk alleles by their reported odds ratio for the disease, and summing this weighted allele score of each variant up into a disease risk score for AD.<br><br>The data of Rotterdam population based cohort Study	19 SNPS	rs429358/rs7412 APOE<br>rs6656401 CR1<br>rs6733839 BIN1<br>rs35349669 INPP5D<br>rs190982 MEF2C<br>rs10948363 CD2AP<br>rs2718058 NME8<br>rs1476679 ZCWPW1<br>rs11771145 EPHA1<br>rs28834970 PTK2B<br>rs9331896 CLU<br>rs10838725 CELF1<br>rs983392 MS4A6A<br>rs10792832 PICALM<br>rs11218343 SORL1<br>rs17125944 FERMT2<br>rs10498633 RIN-SLC24A4<br>rs4147929 ABCA7<br>rs7274581 CASS4	OR

Rodriguez ER, *et al.* 2013	In addition to assessing each individual SNP (Model 1),<br>developed a weighted GRS (Model 2) where the weight used for each SNP is the odds ratio (OR) associated with the risk allele,<br>as opposed the OR associated with the minor allele that was reported in the original GWAS and/or AlzGene database (Bertram et al. 2007).<br>The weighted GRS was calculated by multiplying the number of risk alleles for each SNP (0, 1, or 2) by the weight for that SNP, and then taking the sum across the eight SNPs.<br>Divided the continuous GRS into tertiles and compared risk between them.	8 SNPs	ABCA7 rs3764650,<br>BIN1 rs744373,<br>CD2AP rs9296559,<br>CLU rs1113600,<br>CR1 rs1408077,<br>MS4A4E rs670139,<br>MS4A6A rs610932,<br>PICALM rs3851179	OR
Sims R, *et al*. 2015	Polygenic risk score analysis calculates the collective contribution of common SNPs that show disease association but fail to meet the accepted p value threshold for genome-wide significance, although this does not take into account epistasis between the selected variants. The analysis requires two independent data sets.<br>For the first, result data is sufficient as this data set is used to select the SNPs, the risk score alleles and their genetic effects. The second data set is used to test whether the polygenic risk scores differ in cases and controls and requires the genotypes for each individual. Using the IGAP GWAS data set	21 SNPs	BIN1 rs6733839<br>PICALM rs10792832<br>CLU rs9331896<br>CR1 rs6656401<br>MS4A6A rs983392<br>ABCA7 rs4147929<br>PTK2B rs28834970<br>EPHA1 rs11771145<br>HLA-DRB/DRB1 rs9271192<br>CD2AP rs10948363<br>ZCWPW1 rs1476679<br>NME8 rs2718058<br>SLC24A4/RIN3 rs10498633<br>FERMT2 rs17125944<br>TRIP4 rs74615166<br>INPP5D rs35349669<br>MEF2C rs190982<br>CASS4 rs7274581<br>CELF1 rs10838725<br>TP53INP1 gene-wide<br>IGHV1-67 gene-wide	OR

Table 1. Summary table of previous studies constracting a polygenic risk score (PRS)/ polygenic Hazard score (PHS) in Alzheimer's disease. The most potential group of SNPs used from the GWAS and the method of calculating the score/weighting the polygenic risk score are shown. Odds Ratio (OR) was used to calculate for PRS and Hazard Ration (HR) for PHS used in their respective studies.

PRS: Polygenic risk score

PHS: Polygenic hazard score

SNP: Single nucleotide polymorphism;

OR: Odds Ratio;

HR: Hazard Ratio

ADNI: Alzheimer's Disease Neuroimaging Initiative

IGAP: International Genomics of Alzheimer's Project;

WRAP: Wisconsin Registry for Alzheimer's Prevention;

W-ADRC: Wisconsin Alzheimer 's disease Research Centre;

GERAD consortium: The Genetic and Environmental Risk in AD consortium

GR@ACE: Genome Research at Fundació ACE study

DEGESCO: Dementia Genetics Spanish Consortium

KnightADRC: Knight-Alzheimer's Disease Research Center

DIAN: Dominantly Inherited Alzheimer Network (study at Washington University National NIA-LOAD: Institute on Aging Genetics Initiative for Late-Onset Alzheimer's Disease

ROSMAP: Religious Orders Study and Rush Memory and Aging Project

OATS: Older Australian Twins Study

SydneyMAS: Sydney Memory and Ageing Study

QTIM: Queensland Twin Imaging
